# Label efficient phenotyping for Long COVID using electronic health records

**DOI:** 10.1038/s41746-025-01617-y

**Published:** 2025-07-04

**Authors:** Chuan Hong, Jun Wen, Harrison G. Zhang, Vidul Ayakulangara Panickan, Doris Y. Yang, Alicia W. Chen, Xin Xiong, Xuan Wang, Michele Morris, Sara Morini, Rahul Sangar, Andrew Dey, Malarkodi J. Samayamuthu, Katherine Liao, Clara-Lea Bonzel, Vidisha Tanukonda, Monika Maripuri, Jacqueline Honerlaw, Yuk-Lam Ho, Shyam Visweswaran, Isaac S. Kohane, Kelly Cho, Gabriel Brat, Zongqi Xia, Tianxi Cai

**Affiliations:** 1 Department of Biostatistics and Bioinformatics, Duke University, Durham, NC USA; 2 Department of Biomedical Informatics, Harvard Medical School, Boston, MA USA; 3 Division of Population Health and Data Science, VA Boston Healthcare System, Boston, MA USA; 4 Department of Biomedical Informatics, University of Pittsburgh, Pittsburgh, PA USA; 5 Division of Rheumatology, Inflammation, and Immunity, Brigham and Women’s Hospital, Boston, MA USA; 6 VA Atlanta Healthcare System, Decatur, GA USA

**Keywords:** Viral infection, Statistics

## Abstract

Long COVID poses a significant disease burden globally, but its heterogeneous presentation and unreliable coding practices render it difficult to study. Developing efficient phenotyping algorithms is crucial to enabling risk prediction and effective management of Long COVID. We introduce the LAbel-efficienT Long COVID pHenotyping (LATCH) algorithm, which synthesizes a small number of gold-standard labels and a large, unlabeled dataset with many electronic health record (EHR) features. Both internal validation and external validation demonstrated the superior performance of LATCH over methods using the U09.9 Long COVID EHR code alone. Our downstream analysis revealed a pattern of elevated healthcare utilization due to Long COVID, peaking at and continuing beyond the fourth month following COVID infection. LATCH enhances the classification of Long COVID by fully utilizing both labeled and unlabeled data, providing vital insights into healthcare utilization trends, informing clinical and public health responses to the enduring consequences of COVID-19.

## Introduction

The long-term consequences of the Coronavirus Disease 2019 (COVID-19) remain a major public health concern^1–3^. Long COVID features, also described as “Post-Covid Conditions,” encompass a wide range of often debilitating symptoms arising at least four weeks after the acute infection of SARS-CoV-2^4^. Estimates of Long COVID incidence diverge greatly, and heterogeneous research methods could yield inaccurate capture of Long COVID^1,5–7^. Computable phenotypes based on routine clinical data in the electronic health records (EHR) such as demographic profile, symptoms, laboratory test results, medication prescriptions, procedures, and referral to specialized care can standardize the case capture^8–10^. To better understand the real-world burden of Long COVID and devise public health responses, evidence-based EHR-derived Long COVID models for predicting risk and disease trajectory would be crucial to improve the management of Long COVID^11^.

Studying Long COVID with EHR data is highly challenging, in part due to the lack of reliable code or algorithm that can accurately capture the status and onset time of the condition. The ICD-10 code U09.9, introduced in October 2021^12–16^, has not been consistently implemented across healthcare systems^2,13,17^. In our previous study, based on a chart review validation of 900 COVID-19 patients from three US healthcare systems, we reported an inadequate positive predictive value (PPV) and true positive rate (TPR) of the U09.9 code in classifying Long COVID, underscoring the limitation of using U09.9 as an identifier of Long COVID^18^. Apart from the U09.9 code, published Long COVID phenotypes employ other surrogate features such as earlier SARS-CoV-2 infections^19–21^, clinical terms and symptoms associated with Long COVID^14,20,22–25^, SNOMED clinical terms^12^, records of Long COVID clinic visits^26^, and online survey data^27^, along with diverse algorithms such as rule-based systems and tree-based models. However, all these approaches lack rigorous validation against a chart-reviewed gold-standard label. Moreover, besides leveraging structured data, the use of unstructured clinical narratives through natural language processing (NLP) has been explored by recent studies to uncover additional insights into Long COVID^28–30^. However, these efforts have largely focused on extracting specific symptoms, without fully integrating unstructured data with structured data to identify a comprehensive algorithm to accurately classify Long COVID phenotype.

In this study, we developed a novel LATCH algorithm for identifying Long COVID that delivers four key contributions. First, we circumvent the typical feature dimension restrictions that come with a small set of chart-reviewed gold-standard labels, incorporating the full set of high-dimensional EHR features. Through a multi-step semi-supervised model, we reduce the need for extensive gold-standard labels while still enabling the exploration of complex feature spaces. Second, incorporating information from both structured and unstructured data, our approach improves the accuracy of the Long-COVID phenotype. Third, LATCH addresses temporal variations by differentiating between the “pre-U09.9” and “post-U09.9” periods, reflecting changes in clinical coding and diagnosis practices over time. It also adjusts for the heterogeneity of the initial COVID-19 infection by distinguishing between inpatient and outpatient cases, enhancing the accuracy and applicability of our Long COVID identification. Lastly, the robustness of our algorithm has been validated both internally and externally across two healthcare systems, showcasing its potential for widespread adoption in clinical practice.

## Results

### Study population

**Fig. 1:**
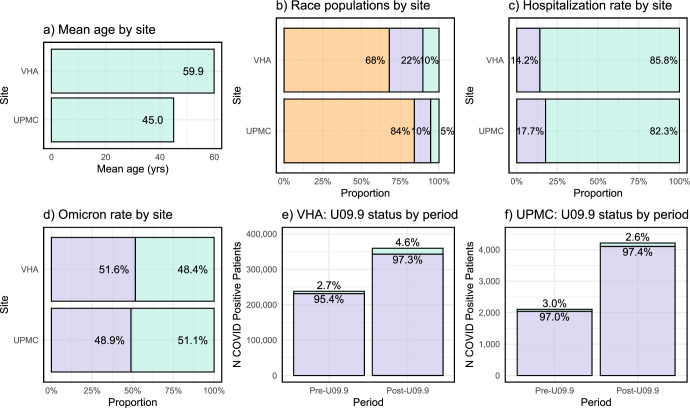
Mean age, race, hospitalization, variant, and the usage of U09.9 across VHA and UPMC. **a** Mean age by site. **b** Race populations by site. Orange—White, Purple—Black or African American, Green—Other race or not reported. **c** Hospitalization rate by site. Purple—Inpatient, Green—Outpatient. **d** Omicron rate by site. Purple—Omicron, Green—Other Variant. **e** VHA: U09.9 status by period. Green—with U09.9, Purple—without U09.9. **f** UPMC: U09.9 status by period. Green—with U09.9, Purple—without U09.9.

The study cohort included a total of 593,283 COVID-19-positive patients from the VHA and 6317 patients from UPMC. Demographic distribution shows variation across the two sites (Fig. 1 and Table 1). The mean age at the time of COVID-19 diagnosis was 59.9 years among VHA patients and 45.0 years among UPMC patients. VHA has a higher percentage of male patients (87.6%) compared to UPMC (42.7%). Black or African American patients comprised 21.7% at the VHA and 10.5% at UPMC. There is a slightly lower proportion of patients at the VHA hospitalized (14.2%) compared to UPMC (17.7%). The analysis of COVID-19 variants among patients revealed a dominant presence of the Omicron variant in both VHA (51.6%) and UPMC (48.9%). In the pre-U09.9 period, VHA had a rate of 2.73% and UPMC had a rate of 3.0% for assigning the U09.9 code (due to back-coding of diagnosis). In the post-U09.9 period, VHA exhibited a rate of 4.63%, while UPMC had a rate of 2.45% for assigning the U09.9 code.

**Table 1:** Patient Demographics at Veterans Health Administration (VHA) and University of Pittsburgh Medical Center (UPMC)

	Covid positive		Covid positive with U09		Covid positive with no U09	
Health system	VHA	UPMC	VHA	UPMC	VHA	UPMC
Total	597,283	6317	23,144	172	574,139	6145
Age at incident COVID diagnosis Mean (SD)	59.9 (16.3)	45.0 (24.5)	61.5 (15.4)	55.1 (17.2)	59.8 (16.4)	44.7 (24.7)
Sex						
Male	523,498 (87.6%)	2699 (42.7%)	19,976 (86.3%)	63 (36.7%)	503,522 (87.7%)	2636 (42.9%)
Female	73,784 (12.4%)	3618 (57.3%)	3168 (13.7%)	109 (63.4%)	70,616 (12.3%)	3509 (57.1%)
Race						
White	405,776 (67.9%)	5311 (84.1%)	16,796 (72.6%)	141 (82.0%)	388,980 (67.8%)	5170 (84.1%)
Black or African American	129,635 (21.7%)	666 (10.5%)	3745 (16.2%)	25 (14.5%)	125,890 (21.9%)	641 (10.4%)
Asian	7680 (1.3%)	65 (1.0%)	256 (1.1%)	2 (1.2%)	7424 (1.3%)	63 (1.0%)
American Indian or Alaskan Native	5188 (0.9%)	39 (0.6%)	239 (1.0%)	0 (0.0%)	4949 (0.9%)	39 (0.6%)
Native Hawaiian or Pacific	6680 (1.1%)	1 (0.02%)	265 (1.1%)	0 (0.0%)	6145 (1.1%)	1 (0.02%)
Not Respond	42,324 (7.1%)	235 (3.7%)	1843 (8.0%)	4 (2.3%)	40,481 (7.1%)	231 (3.8%)
Period						
Pre-U09.9	237,850 (39.8%)	2111 (33.4%)	6488 (28.0%)	69 (40.1%)	231,362 (40.3%)	2042 (33.2%)
Post U09.9	359,433 (60.2%)	4206 (66.6%)	16,656 (72.0%)	103 (59.9%)	342,777 (59.7%)	4103 (66.8%)
Hospitalization						
Inpatient	84,826 (14.2%)	1119 (17.7%)	6413 (27.7%)	53 (30.8%)	78,413 (13.7%)	1066 (17.3%)
Outpatient	512,457 (85.8%)	5198 (82.3%)	16,731 (72.3%)	119 (69.2%)	495,726 (86.3%)	5079 (82.7%)
Variant						
Alpha	23,156 (3.9%)	305 (4.8%)	599 (2.6%)	17 (9.9%)	22,557 (3.9%)	288 (4.7%)
Delta	91,360 (15.3%)	1284 (20.3%)	5504 (23.8%)	67 (39.0%)	85,856 (15.0%)	1217 (19.8%)
Omicron	308,460 (51.6%)	3091 (48.9%)	12,953 (56.0%)	45 (26.2%)	295,507 (51.5%)	3046 (49.6%)
Other	174,307 (29.2%)	1637 (25.9%)	4088 (17.7%)	43 (25.0%)	170,219 (29.6%)	1594 (25.9%)

### Internal validation at the VHA

**Fig. 2:**
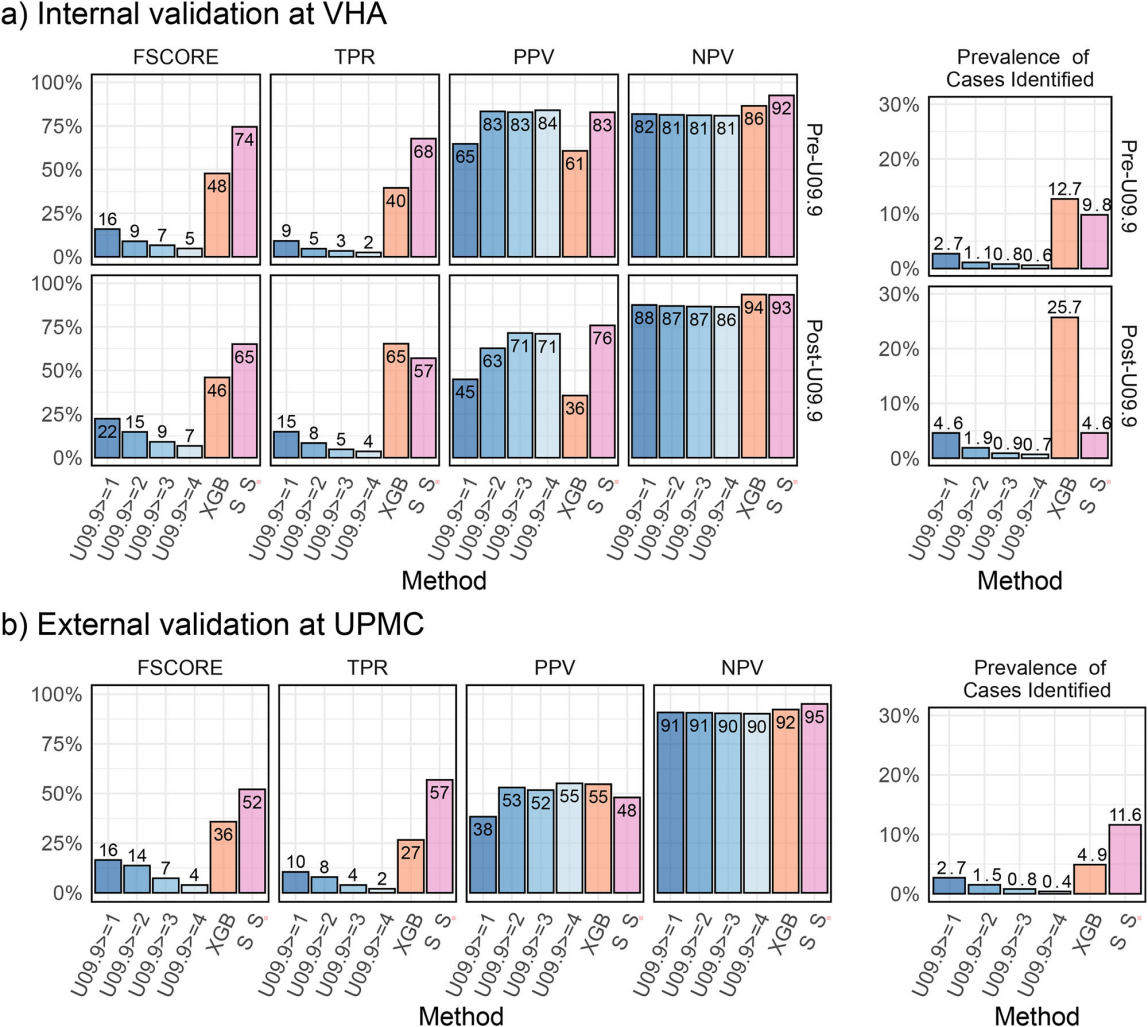
Classification performance of the proposed semi-supervised method (SSL) vs benchmarking methods. Benchmark methods for comparison were U09.9 counts greater or equal to 1, 2, 3 and 4, and unsupervised XGBoost (XGB). For all methods shown, F-Score, TPR, PPV, NPV and prevalence of cases were identified through evaluation against the gold-standard chart review labels using WHO-1 definition at (**a**) VHA and (**b**) UPMC. VHA Health Administration, UPMC University of Pittsburgh Medical Center, XGB XGBoost tree models, TPR True Positive Rate, PPV Positive Predictive Value, NPV Negative Predictive Value.

Figure 2a illustrates the performance of different phenotyping methods against the WHO-1 Long COVID definition across pre- and post-U09.9 time frames. During pre-U09.9, the binary presence of the U09.9 code resulted in an F-score of 15.9%, TPR of 9.1%, and PPV of 64.7%. Other rule-based methods using the U09.9 code with adjusted thresholds exhibited F-score ranging from 4.8% to 8.9%, TPRs from 2.5% to 4.7%, and PPVs from 82.9% to 84%. In contrast, the unsupervised XGBoost model showed a higher F-score (47.8%) and TPR (39.5%) but with a lower PPV (60.7%). The proposed semi-supervised algorithm demonstrated a notable improvement with an F-score of 75.4%, TPR of 67.7%, and PPV of 82.8%. Similarly, during post-U09.9, the proposed algorithm surpassed methods using the U09.9 code alone, marked by a higher F-score (65.1% vs. 22.3%), TPR (57.0% vs. 14.9%), and PPV (75.8% vs. 44.9%).

For the WHO-2 definition (Supplementary Fig. 2), all methods exhibited systematically lower PPVs compared to the WHO-1 definition, due to the more stringent criteria, while similarly to the WHO-1 definition, the proposed algorithm consistently outperformed others in detecting Long COVID cases against the WHO-2 definition.

For both the WHO-1 and the WHO-2 definitions across all model versions, the inputs to the semi-supervised step have comparable feature importance. Shapley feature importance values for the top 10 inputs to the unsupervised steps and all inputs to the semi-supervised step are summarized in Supplementary Fig. 4.

### External validation at UPMC

Figure 2b shows the PheCode-only external validation performance of various phenotyping algorithms at UPMC, against the *WHO-1* and *WHO-2* definitions. Similar to the findings seen at the VHA, methods leveraging the U09.9 code showed high PPVs but markedly lower TPRs. Under the *WHO-1* definition, the binary presence of the U09.9 code yielded an F-score of 16.5%, TPR of 10.5%, and a PPV of 38.3%. Rule-based methods using the U09.9 code had F-scores ranging from 3.9% to 13.7%, TPRs from 2.0% to 7.9%, and PPVs from 51.7% to 55.1%. The unsupervised XGBoost approach surpassed the U09.9 code-based methods, achieving an F-score of 35.8%, TPR of 26.6% and a PPV of 54.7%. The proposed semi-supervised algorithm outperformed other methods by attaining a higher F-score (52.1%), TPR (56.9%), and PPV (48%). For the WHO-2 definition, similar patterns were observed, indicating the robustness and enhanced detection capabilities of the proposed method across different clinical definitions. The U09.9 count is the most important feature for both the model trained with WHO-1 labels and the model trained with WHO-2 labels, as measured by Shapley value (0.250 and 0.197, respectively). Shapley feature importance values for the top 10 inputs to the unsupervised steps and all inputs to the semi-supervised step are summarized in Supplementary Fig. 4.

### Temporal trend analysis of post-infection healthcare utilization

**Fig. 3:**
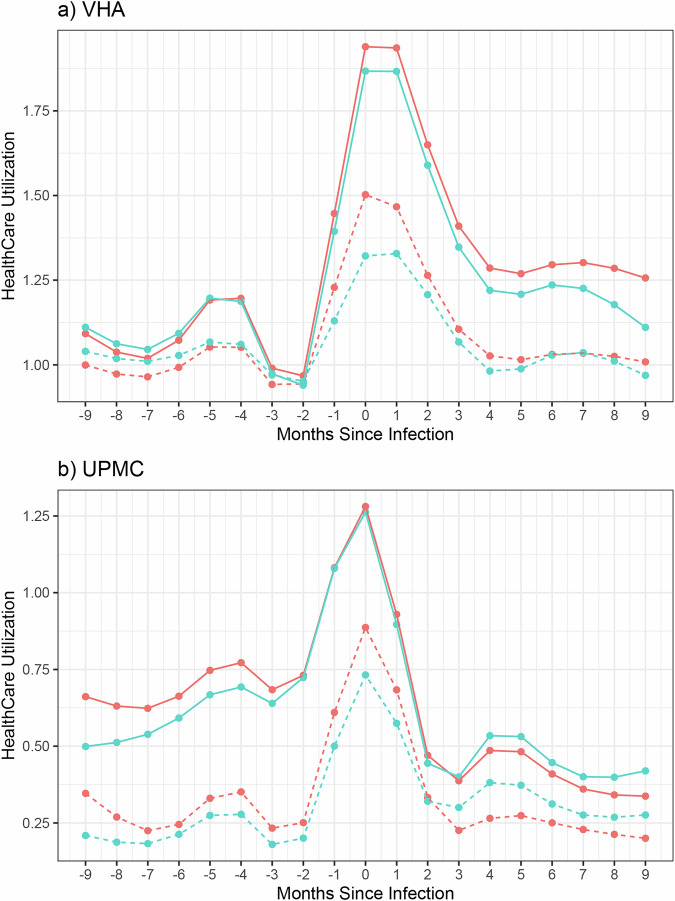
Pre- and post-infection healthcare utilization trend among patients identified as Long COVID cases vs. identified as Long COVID controls. The healthcare utilization data shown is stratified by different time periods. Healthcare utilization is measured as the total number of days each month with at least one PheCode observed in the EHR. Each data point represents the number of days in that given month that at least one PheCode was entered into the EHR, averaged across patients. **a** Pre- and post-infection healthcare utilization trend at VHA; **b** Pre- and post-infection healthcare utilization trend at UPMC. Solid line Long COVID cases, Dotted line Long COVID controls, Red Pre-U09.9 Period, Blue Post-U09.9 period. VHA Veterans Health Administration, UPMC University of Pittsburgh Medical Center.

Figure 3 displays the temporal trend analysis of pre- and post-infection healthcare utilization over pre-U09.9 (marked by a red line) and post-U09.9 (blue line). Individuals identified as Long COVID-positive (marked by solid lines) by the proposed phenotyping algorithm consistently required more healthcare resources than those without Long COVID (marked by dotted lines) across both periods, highlighting the prolonged impact of Long COVID. A similar healthcare utilization curve shape is evident across Long COVID status and period of infection, namely a small spike 4–5 months before the large spike in the month of infection, followed by another small spike 4–5 months after infection. Notably, there was an increased healthcare usage among Long COVID cases compared to those who did not have Long COVID even before their COVID-19 diagnosis, in line with a previous study that included baseline healthcare utilization^31^. This was possibly due to a lag in infection detection until enough evidence was accumulated, and the increased risk of Long COVID due to preexisting conditions that necessitated elevated healthcare usage prior to COVID-19 infection. Baseline healthcare utilization was significantly higher at the VHA as compared to UPMC, which is reasonable given the difference in patient populations served and healthcare system structure between the VHA and UPMC. Following infection, there’s a significant initial decrease in healthcare usage until the 4th month at VHA and the 3rd month at UPMC. Similar findings were observed when stratified by COVID-19 variants (Supplementary Fig. 3), suggesting that the disparate effects of Long COVID persist regardless of the variant of the initial infection.

## Discussion

In this study, we propose a novel semi-supervised phenotyping algorithm trained on a large dataset from the VHA. This approach effectively leveraged a combination of expert-annotated gold-standard labels and a vast array of EHR features, including both structured data and NLP-processed unstructured data. Through internal validation within VHA and partial external validation at UPMC, we demonstrated that our semi-supervised algorithm achieved both higher PPV and TPR values, as compared to several benchmark methods using various knowledge extraction methods, data types, and levels of supervision. These comparisons show that our method benefits from the combination of structured data and named-entity recognition symptom extraction from unstructured data in a semi-supervised manner. Our downstream analysis based on the phenotyping classification reveals significant trends in healthcare utilization that extend well beyond the acute phase of COVID-19, with Long COVID-positive patients consistently presenting higher utilization rates compared to their Long COVID-negative counterparts. This trend was particularly pronounced in the early months post-infection, followed by a gradual decline. Interpreting these results suggests a marked burden on healthcare systems due to Long COVID, highlighting the need for continued patient support and resource allocation long after the initial infection.

The first strength of this study lies in its innovative methodological approach, combining the depth of chart-reviewed data with the breadth of EHR data to inform phenotyping. Previous Long COVID phenotyping studies have compromised either gold-standard labels or the size of the feature space. Our algorithm avoids this tradeoff by using two stages of training: the first training stage allows us to explore the full EHR feature space by using U09.9 as a surrogate label while the second training stage fine-tunes the predictions from the first stage using a smaller set of gold-standard labels. Feature importance, as measured by Shapley value in Supplementary Fig. 4, provides some interpretability for both stages of training. The second strength of this study comes from the feature curation step. In particular, we integrated both structured and unstructured EHR data, in contrast to previous studies focusing only on one type of data. The third strength of this study is the high performance according to a variety of metrics achieved by our phenotyping algorithm. Accurate Long COVID phenotype labels are crucial to downstream studies of the disease, with our analysis of healthcare utilization being one such example. Genome-wide association studies are another example of a downstream application that requires accurate phenotype labels in order to detect signals. Improving clinical management of this new disease depends on downstream studies of risk prediction and disease trajectory, such as GWAS and studies of healthcare utilization.

In the current implementation, we choose the lightweight NER-based information extraction to represent EHR notes based on counts of candidate features due to their computational efficiency and transportability across health systems. Regardless of the NLP technique used, the core contribution of our pipeline remains the design of our semi-supervised model that synthesizes codified and NLP data. Acknowledging that more powerful language models can potentially capture more detailed information, we conducted a comprehensive comparison with a more advanced Bert-based approach (ClinicalBERT^32^). As shown in Supplementary Table 3, both the NER-based and ClinicalBERT approaches show similar performance across various evaluation scenarios. However, the NER method offers significant advantages in terms of computational efficiency and interpretability.

Limitations exist, however, particularly with regard to the data used in this study. Our algorithm was trained with data from a single healthcare system, which may not be generalizable across different populations or healthcare settings. Several limitations also arise from the external validation process at UPMC. While we did internally validate our results at the VHA, external validation occurred at a single healthcare system with limited gold-standard labels. Moreover, we externally validated only the structured data component of our algorithm and did not differentiate between pre- versus post-U09.9 period of infection. As evidenced by the multiple definitions of Long COVID used to curate gold-standard labels in this study, the evolving disease definition introduces uncertainty into the phenotyping algorithm, particularly its ability to identify weaker cases of Long COVID or those without a U09.9 code. Despite these limitations, especially the lower external validation performance, our algorithm still holds practical value for studying Long COVID. Specifically, it significantly narrows the pool for identifying positive cases, and the strong NPV supports the effective identification of control cases. Even with the UPMC results, our proposed semi-supervised algorithm achieves a sensitivity of 56.9%, a PPV of 48%, and an NPV of 95.1%, compared to U09.9, which yields only 10.5% sensitivity and 38.3% PPV. While misclassification in the SSL-based approach leads to some power loss relative to studies using a gold standard phenotype definition, it still supports reasonably powered association studies. For example, using the SSL-defined phenotype allows for the detection of smaller effect sizes with a given sample size compared to using the gold standard phenotype. In contrast, relying solely on the U09.9 code to define Long COVID requires detecting significantly larger effect sizes to achieve the same statistical power. These results highlight that, despite its suboptimal accuracy, the SSL algorithm-defined Long COVID significantly advances the potential for conducting large-scale studies on Long COVID.

In addition to exploring better integration of the strengths of structured and unstructured EHR data, particularly under the more noise susceptible WHO-1 definition, future studies should aim to integrate additional data sources, such as patient-generated health data from wearables and patient-reported outcome measures, to enrich the phenotyping algorithms further and strengthen the TPR and F-scores. Long-term monitoring and follow-up of patients are crucial to understanding the evolving nature of Long COVID and its implications on individuals’ health trajectories. For instance, the similarity in healthcare utilization curve shape among patients with Long COVID and patients with COVID, particularly the dip starting at month 4 followed by a slight uptick in months 5 and 6, is a trend that warrants deeper investigation in the future. Future studies could also explore the types of healthcare utilization and the reasons behind the healthcare utilization trends, ranging from the medical need to the chilling effect of the pandemic on hospital visits to the reduced availability of healthcare due to the pandemic-related strain on the healthcare system.

The utility of this research extends into clinical decision-making and public health policymaking. In this study, we are interested in developing a method for computationally determining the Long COVID status. The ability to computationally and accurately determine Long COVID status of patients from the EHR is crucial for conducting Long COVID studies of clinical relevance. Accurately classified long-COVID phenotype can facilitate a wide range of clinical and translational studies such as identifying risk factors for developing Long COVID, assessing the effect of COVID vaccine or treatment on the risk of PASC, performing genome-wide association studies (GWAS) to identify genomic variants associated with the risk of developing PASC as well as drug-repurposing studies to identify potential drugs for reducing the risk of PASC. Accurate phenotype labels are crucial to ensure power and mitigate bias in these studies. By providing a more accurate tool for identifying Long COVID in future studies, physicians will be able to better tailor their care strategies to the needs of Long COVID patients. For public health officials, these insights are invaluable for planning purposes, enabling the allocation of resources to where they are most needed and informing policy development to support the long-term well-being of COVID-19 survivors. As the pandemic continues to evolve, so too must our strategies for managing its aftermath, with research such as this study contributing critical knowledge to that end.

## Methods

### Study design and setting

**Fig. 4:**
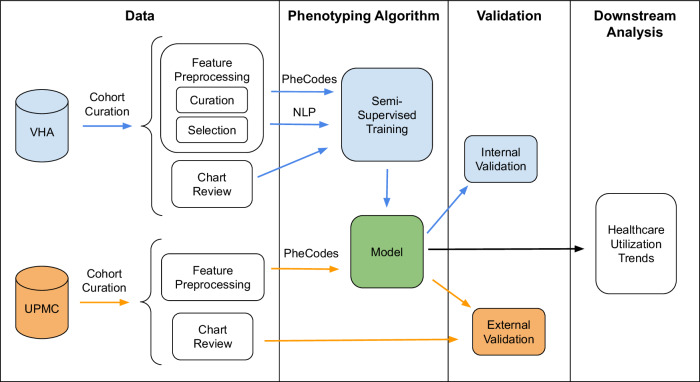
Overall workflow for development of LATCH phenotype. Phenotype development consisted of the curation of COVID-19 patient cohorts from two healthcare systems, the subsequent development and validation phases of our novel phenotyping algorithm, and the downstream practical application in examining healthcare utilization trends associated with Long COVID. LATCH - LAbel-efficienT Long COVID pHenotyping.

The overall workflow (Fig. 4) includes cohort curation of patients with COVID-19 from two healthcare systems, the development and validation of the proposed phenotyping algorithm, followed by an illustrative downstream clinical application (i.e., healthcare utilization trends related to Long COVID).

### Data source

The Consortium for Clinical Characterization of COVID-19 by EHR (4CE) is an international consortium for data-driven studies pertaining to COVID-19 and Long COVID^33^. Two healthcare systems from the 4CE Consortium contributed data and chart review results for the current study: the Veterans Health Administration (VHA) and the University of Pittsburgh Medical Center (UPMC)^34–36^. The VHA is the largest integrated healthcare system in the United States, with 171 medical centers, and UPMC is a Pittsburgh-based healthcare system with 43 hospitals. Over 15 million patients receive care at these two healthcare systems. The Institutional Review Boards (IRBs) at each of the participating healthcare systems approved the study (MVP: Supported by the Million Veteran Program (MVP000)—Central IRB 10-02; Phenotyping Protocol: Boston IRB 3097; Innovative Analytics: Central IRB 18–38; UPMC: STUDY20070095). Waivers of informed consent and waivers of HIPAA Authorization were received for these data only analysis studies.

We used data from VHA to train and internally evaluate the phenotyping algorithm, and those from UPMC to externally validate the component of the algorithm trained on structured data. At each healthcare system, we curated the cohort utilizing the same strategy. (1) *Inclusion criteria and index date*. The study cohort comprised patients who were either assigned at least one ICD-10 code of U07.1 (“COVID-19, virus identified”) or had confirmed positive results from a SARS-CoV-2 reverse transcription polymerase chain reaction (PCR) test, within the period from March 1, 2020, to September 30, 2022 for VHA, and to March 31, 2023 for UPMC. For each patient, the index date was set to the date of their first U07.1 code or positive PCR test for SARS-CoV-2. (2) *Inpatient vs. outpatient*. Patients meeting the inclusion criteria were further grouped as hospitalized (inpatient) or non-hospitalized (outpatient) depending on their hospital admission status within a window of 7 days before to 14 days after the index date. (3) Pre- vs. post-U09.9. To assess the potential role of the introduction of the U09.9 code on Long COVID phenotyping, we divided the cohort into two periods: pre- and post-U09.9. The infection cutoff date was set to September 1, 2021, to accommodate a lag window of up to 30 days for coding Long COVID, corresponding with the introduction of the U09.9 code in October 2021.

### Long COVID definition and chart review process

Validation of Long COVID through chart review adhered to the World Health Organization (WHO) definition of Long COVID^37,38^, following a VHA-developed protocol^39^ with insights from 4CE Consortium. Eleven common Long COVID symptoms were identified into a “core” symptom cluster^40–43^, with additional symptoms identified into an “extended” cluster by disease domain (e.g., cardiovascular, respiratory). Chart reviews were conducted on sampled patients with over six months of post-infection clinical notes and having at least one U09.9 code or new onset Long COVID related ICD code. This ensured documentation of symptom onset and duration aligning with Long COVID definitions (Supplementary Fig. 1). Descriptive statistics for the chart review cohort can be found in the previously published study by Maripuri et al.^39^. A case was classified under less stringent criteria (WHO-1) if a single core symptom persisted for more than 60 days post-infection, whereas more stringent criteria (WHO-2) required at least two new symptoms (either two core or one core plus one extended) to persist for more than 60 days post-infection.

Domain experts chart reviewed clinical notes up to one year prior to the initial acute COVID-19 episode, excluding any symptoms present before or concurrently with the acute phase as the new onset. At the VHA, 474 patients were reviewed, including 332 randomly selected patients with U09.9 code and 142 patients without. At UPMC, 178 were reviewed, including 74 randomly selected patients with U09.9 code and 104 without.

### Data process, feature curation and selection

Data from the VHA comprised both structured and unstructured types, while those from UPMC was solely structured data. For *structured data*, we rolled all ICD-10 diagnosis codes to one-digit level PheCodes^44^ to capture broader diagnoses, intentionally omitting multi-level PheCodes. For *unstructured data*, we extracted Long COVID-related concepts as Concept Unique Identifiers (CUIs) using our established NLP pipeline^45,46^. This involved applying named entity recognition (NER) to eight PubMed review articles and seven online knowledge databases to construct comprehensive CUI dictionaries. Following this, NLP was employed to process the narrative notes and extract the CUIs in the dictionary. We curated PheCode and NLP data into two types of features: the count of new-onset features post-COVID-19 infection per patient and the duration (in months) those features were observed. Following the exclusion of features with over 99% zero occurrences, we employed surrogate-assisted feature selection^47^ to define our candidate feature set.

### Semi-supervised phenotyping

**Fig. 5:**
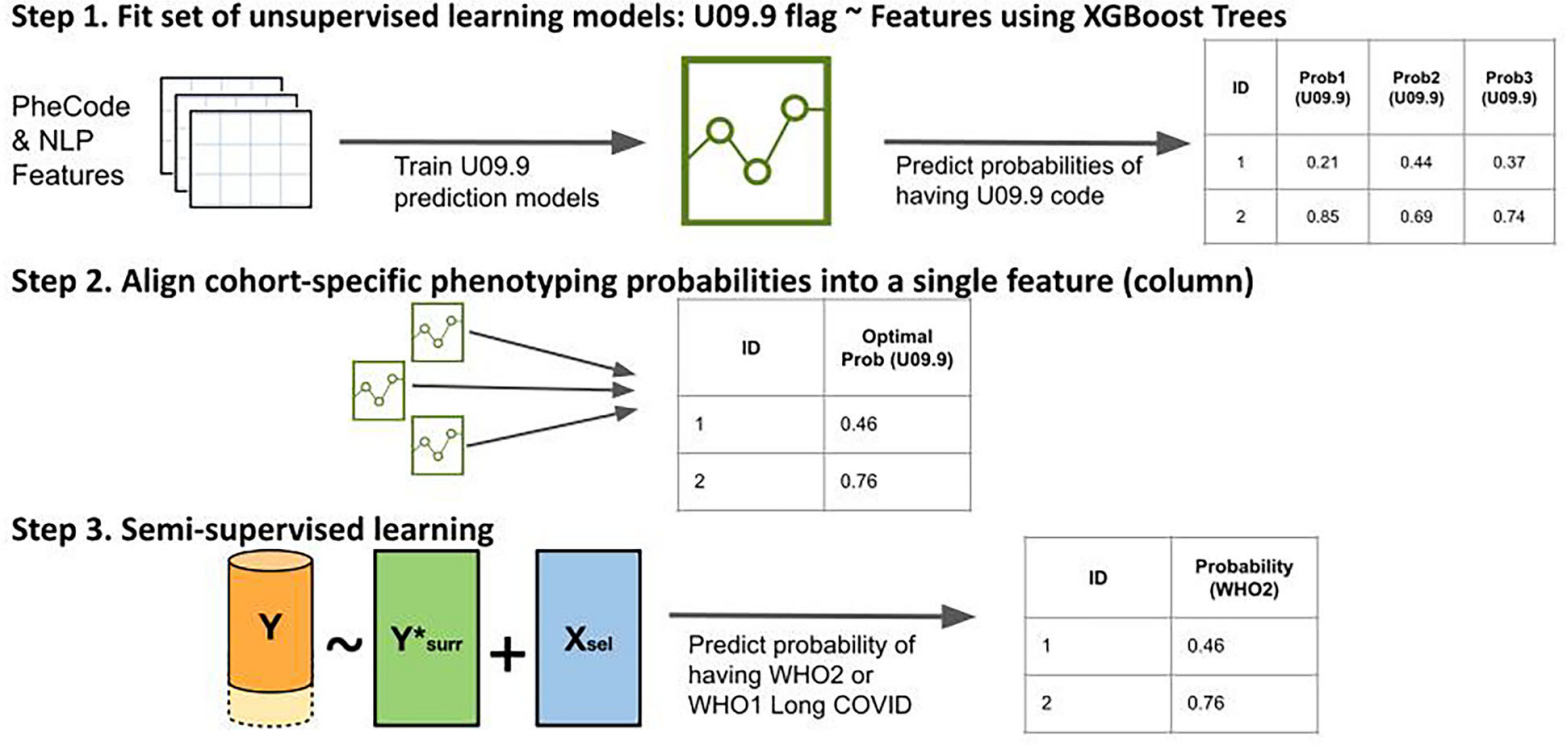
Three-step semi-supervised LATCH phenotyping. The steps for semi-supervised LATCH phenotyping are 1) unsupervised models without using gold-standard chart review labels, 2) alignment of cohort-specific probabilities and 3) supervised model, incorporating gold-standard chart review labels. LATCH LAbel-efficienT Long COVID pHenotyping. NLP Natural Language Processing.

Utilizing VHA data, we developed a three-step semi-supervised LATCH phenotyping algorithm as illustrated in Fig. 5, where we initially built unsupervised models without using gold-standard chart review labels, and finally built a supervised model, incorporating gold-standard chart review labels.

1. *Unsupervised XGBoost*. We trained a set of XGBoost tree models^48^ to classify the presence of U09.9, as a binary noisy label for the true Long COVID status, using curated candidate features from EHR data. Note that U09.9 data was retrospectively available even for the pre-U09.9 cohort due to back-coding procedures well established at institutions like the VHA and UPMC. Using the noisy label of U09.9 allowed for training with the full study cohort without gold-standard labels, and the XGBoost tree method was chosen to accommodate high dimensional data and to capture non-linear associations. The XGBoost models were tailored to specific sub-cohorts based infection period (i.e., pre-U09.9 or post-U09.9), hospitalization type. For each sub-cohort, models were trained with either 1) PheCode features only, or 2) combined PheCode and NLP features. This resulted in a set of XGBoost probabilities tailored to specific sub-cohorts and data types.
2. *Alignment of cohort-specific probabilities*. We consolidated the XGBoost probabilities specific to each sub-cohort into a singular feature, assigning one probability per patient. Each patient’s final XGBoost probability was chosen from a model trained on the subcohort that matched the patient, based on infection period (i.e., pre-U09.9 or post-U09.9) and type of hospitalization. Within each sub-cohort, U09.9 status (presence or absence of code) determined which data type model was used: patients with U09.9 absent were assigned probability from the model trained with both PheCode and NLP features, patients with U09.9 present were assigned probability from the model trained with PheCode features only.
3. *Supervised logistic regression model*. Employing logistic regression, we used the chart-review cohort to regress the gold-standard label against the binary indicator for period, the unified XGBoost probability, and logarithm of U09.9 code counts to refine patient-level Long COVID status classification.

### Methods for comparison and evaluation metrics

We compared our semi-supervised LATCH algorithm against benchmark models such as binary U09.9 code presence, rule-based phenotypes at varying U09.9 code counts (e.g., ≥ 2, 3, 4), and unsupervised XGBoost-only, unsupervised XGBoost-only using only structured data, and the proposed model using only structured data. The different model architectures of the proposed method and benchmark models are summarized in Supplementary Table 2. Performance metrics, including area under the receiver operating characteristic curve (AUROC), F-score, TPR, PPV, and the proportion of Long COVID identified among COVID-19 patients, were evaluated against gold-standard chart review labels across periods (pre-U09.9, post-U09.9) and against different Long COVID definitions (WHO-1, WHO-2). For internal validation of the proposed method using VHA data, we conducted 10-fold cross-validation due to its reliance on labeled data for training and evaluation. To improve model interpretability, particularly for the unsupervised portion of our model, we also calculated Shapley values for feature importance.

### External validation

Beyond internal validation, we assessed the generalizability of the proposed algorithm through external validation on UPMC data, focusing on the PheCodes only model due to the absence of NLP features for UPMC patients. We used the same benchmarks and evaluation metrics as previously detailed. However, we did not differentiate based on time periods because of fewer gold-standard labels at UPMC compared to VHA.

### Downstream clinical application: temporal trend analysis of pre- and post-infection healthcare utilization

We demonstrate a proof of concept for a downstream clinical application of our method, by comparing pre- and post-infection healthcare utilization between patients identified as Long COVID-positive and Long COVID-negative in order to understand the healthcare impact of Long COVID. In contrast to existing studies relying on rule-based or survey data to identify Long COVID cases, we use the proposed computable phenotypes^31,49–52^. Moreover, our analysis uniquely provides a month-by-month analysis, including the pre-infection period, of the degree of healthcare utilization. Using a longitudinal mixed-effects model, we analyzed healthcare utilization as the monthly total number of days with any PheCodes observed in the EHR, considering both fixed and random effects. The model includes variables such as period (pre-U09.9 vs post-U09.9), time in months pre- and post-infection, logarithm of baseline PheCode counts, and patient ID for random effects. Nonlinear temporal trends were captured using spline functions at the 2nd, 4th, and 6th months post-infection.

## Supplementary information

Supplemental Material

## Data Availability

The visit level data set we used to build the model is not shareable due to privacy constraints. The trained models and summary statistics are available at https://github.com/celehs/PASC.

## Code availability

The codes for model building and data analysis are available at https://github.com/celehs/PASC.
