# Supplementary material for: Label efficient phenotyping for Long COVID using electronic health records
Source: NPJ Digit Med. 2025 Jul 4;8:405. doi: 10.1038/s41746-025-01617-y (PMC12227522; doi:10.1038/s41746-025-01617-y)

SUPPLEMENTARY

Supplementary Table 1. Comparison of phenotyping metrics over different periods at VHA.

Number of observations (N), area under the receiver operating characteristic curve (AUC), F1 score (Fscore), false positive rate (FPR), true positive rate (TPR), positive predictive value (PPV), negative predictive value (NPV), and the proportion of positive cases identified (P.POS).

Label	Period	N	Method	AUC	Fscore	FPR	TPR	PPV	NPV	P.POS
WHO1	Pre-U09.9	237850	U09.9>=1	54.0	15.9	1.2	9.1	64.7	81.8	2.7
			U09.9>=2	54.0	8.9	0.2	4.7	83,3	81.3	1.1
			U09.9>=3	54.0	6.6	0.2	3.4	82.9	81.1	0.8
			U09.9>=4	54.0	4.8	0.1	2.5	84.0	80.9	0.6
			XGB	62.4	47.8	6.2	39.5	60.7	86.5	12.7
			**SSL**	**62.4**	**74.5**	**3.4**	**67.7**	**82.8**	**92.5**	**9.8**
	Post-U09.9	359433	U09.9>=1	56.0	22.3	3.0	14.9	44.9	87.5	4.6
			U09.9>=2	56.0	14.8	0.8	8.4	62.7	86.9	1.9
			U09.9>=3	56.0	9.1	0.3	4.8	71.4	86.6	0.9
			U09.9>=4	56.0	6.8	0.2	3.6	71.0	86.4	0.7
			XGB	73.7	46.0	19.3	65.3	35.6	93.5	25.7
			**SSL**	**73.8**	**65.1**	**3.0**	**57.0**	**75.8**	**93.3**	**4.6**
WHO2	Pre-U09.9	237850	U09.9>=1	56.3	21.3	1.7	14.2	42.9	92.7	2.7
			U09.9>=2	56.3	14.2	0.5	8.1	60.4	92.3	1.1
			U09.9>=3	56.3	11.1	0.3	6.1	62.9	92.2	0.8
			U09.9>=4	56.3	7.8	0.2	4.2	60.0	92.1	0.6
			XGB	72.2	48.6	2.9	42.5	56.8	94.9	6.2
			**SSL**	**72.4**	**49.7**	**3.5**	**45.8**	**54.2**	**95.2**	**7.0**
	Post-U09.9	359433	U09.9>=1	54.6	16.5	3.9	12.9	22.9	92.5	4.6
			U09.9>=2	54.6	13.0	1.3	8.0	34.9	92.3	1.9
			U09.9>=3	54.6	9.4	0.6	5.2	45.2	92.1	0.9

U09.9>=4	54.6	8.1	0,4	4.4	51.6	92.1	0.7
XGB	81.4	34.0	21.7	70.2	22.4	96.7	25.7
**SSL**	**81.4**	**55.9**	**3.9**	**55.6**	**56.1**	**96.0**	**4.6**

Supplementary Table 2.

Model and benchmark methods summary.

Comparison of the level of model supervision and input features of the proposed semi-supervised method (SLL) vs. benchmarking methods.

	Model Supervision		Features	
Model	Unsupervised	Semi-supervised	Structured Data (incl. U09.9 counts)	Unstructured Data
Rule-based*	x		x (U09.9 only)	
XGB all*	x		x (all)	x (NER)
XGB Structured Data Only*			x (all)	
XGB - NLP using ClinicalBert*		x	x (all)	x (embedding)
XGB - NLP using NER*	x		x (all)	x (NER)
SSL Structured Data Only*		x	x (all)	
**SSL - NLP using Clinical Bert**			**x (all)**	**x (embedding)**
**SSL - NLP using NER**		**x**	**x (all)**	**x (NER)**

*baseline models; XGB NLP using ClinicalBert were conducted on a 5000 subsample randomly selected from the cohort to reduce computational cost.

Supplementary Table 3.

Performance of model and benchmark methods.

Comparison of phenotyping metrics between proposed SSL model and select benchmark methods: structured data only models and embedding based NLP. Area under the receiver operating characteristic curve (AUC), false positive rate (FPR), true positive rate (TPR), positive predictive value (PPV), and negative predictive value (NPV).

			FPR set to 0.05		FPR set to 0.10	
**Label**	**Method**	**AUC***	**TPR**	**PPV**	**TPR**	**PPV**
WHO2	XGB - Structured Data Only	0.66	0.20	0.62	0.26	0.51
	XGB - NLP using ClinicalBert	0.70	0.21	0.57	0.28	0.47
	XGB - NLP using NER	0.71	0.21	0.59	0.27	0.48
	SSL - Structured Data Only	0.76	0.28	0.70	0.46	0.65
	SSL - NLP using ClinicalBert	0.78	0.30	0.66	0.49	0.61
	**SSL - Proposed Model**	**0.78**	**0.29**	**0.66**	**0.46**	**0.61**
WHO1	XGB - Structured Data Only	0.70	0.18	0.76	0.26	0.68
	XGB - NLP using ClinicalBert	0.67	0.18	0.76	0.25	0.70
	XGB - NLP using NER	0.71	0.18	0.76	0.26	0.68
	SSL - Structured Data Only	0.79	0.38	0.87	0.46	0.79
	SSL - NLP using ClinicalBert	0.74	0.17	0.75	0.36	0.77
	**SSL - Proposed Model**	**0.79**	**0.37**	**0.87**	**0.45**	**0.79**

**Weighting was not used to account for the sampling of chart review patients to create a direct comparison within the chart review cohort. Additionally chart review patients were screened with an NPV filter (NPV > 0.85 for WHO1, NPV > 0.95 for WHO2) based on XGB score, in order to create evaluation label sets with higher prevalence of true cases.*

Supplementary Figure 1.

Patient sampling strategy for chart review.

Sampling was stratified between patients with and without the U09.9 code to include a wide range of potential Long COVID scenarios. Additionally for VHA, patients were sampled from Pre-U09.9 and Post-U09.9 periods of index infection date to represent a comprehensive variety of cases. a) Patient sampling strategy for chart review flowchart. b) Summary of VHA sampling. c) Summary of all VHA patients. EHR-Electronic Health Records, ICD-International Classification of Diseases, PCR - Polymerase Chain Reaction, VHA – Veterans Health Administration, and UPMC – University of Pittsburgh Medical Center.

A) Sampling by U09.9 and new onset Long COVID symptom ICD codes

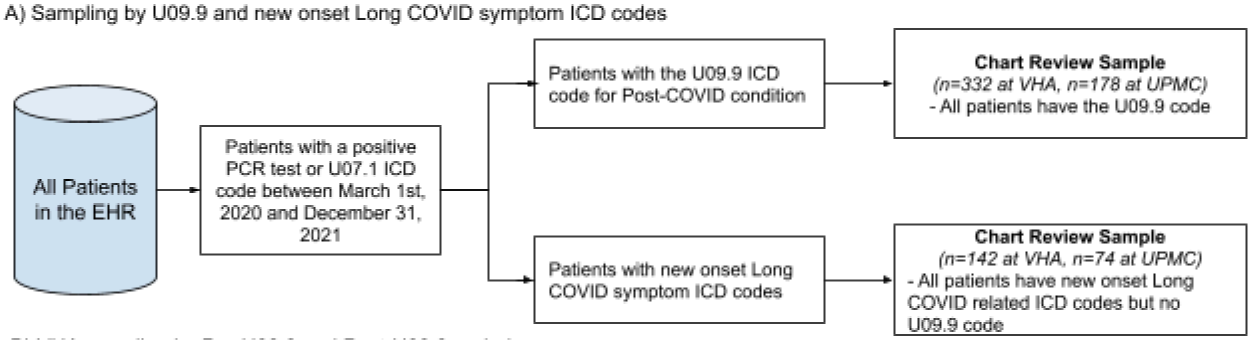

B) VHA sampling by Pre-U09.9 and Post-U09.9 period

**VHA patients with U09.9, n=332**	
Sampled with no period restriction	232
Sampled from Post-U09.9 period	100

**VHA patients with new onset Long COVID symptom code, n=142**	
Sampled with no period restriction	92
Sampled from Pre-U09.9 period	50

C) VHA patients by Pre-U09.9 and Post-U09.9 period

**VHA patients with U09.9, n=332**	
Pre-U09.9 period	116
Post-U09.9 period	216

**VHA patients with new onset Long COVID symptom code, n=142**	
Pre-U09.9 period	113
Post-U09.9 period	29

Supplementary Figure 2.

Classification performance of the proposed semi-supervised method (SSL) vs benchmarking methods, using WHO-2 definition of Long COVID.

Benchmark methods for comparison were U09.9 counts greater or equal to 1, 2, 3 and 4, and unsupervised XGBoost (XGB). For all methods shown, F-Score, TPR, PPV , NPV and prevalence of cases were identified through evaluation against the gold-standard chart review labels using WHO-2 definition. a) Internal validation at VHA. b) External validation at UPMC. VHA - Veterans Health Administration, UPMC - University of Pittsburgh Medical Center, XGB - XGBoost tree models, TPR - True Positive Rate, PPV - Positive Predictive Value, NPV - Negative Predictive Value

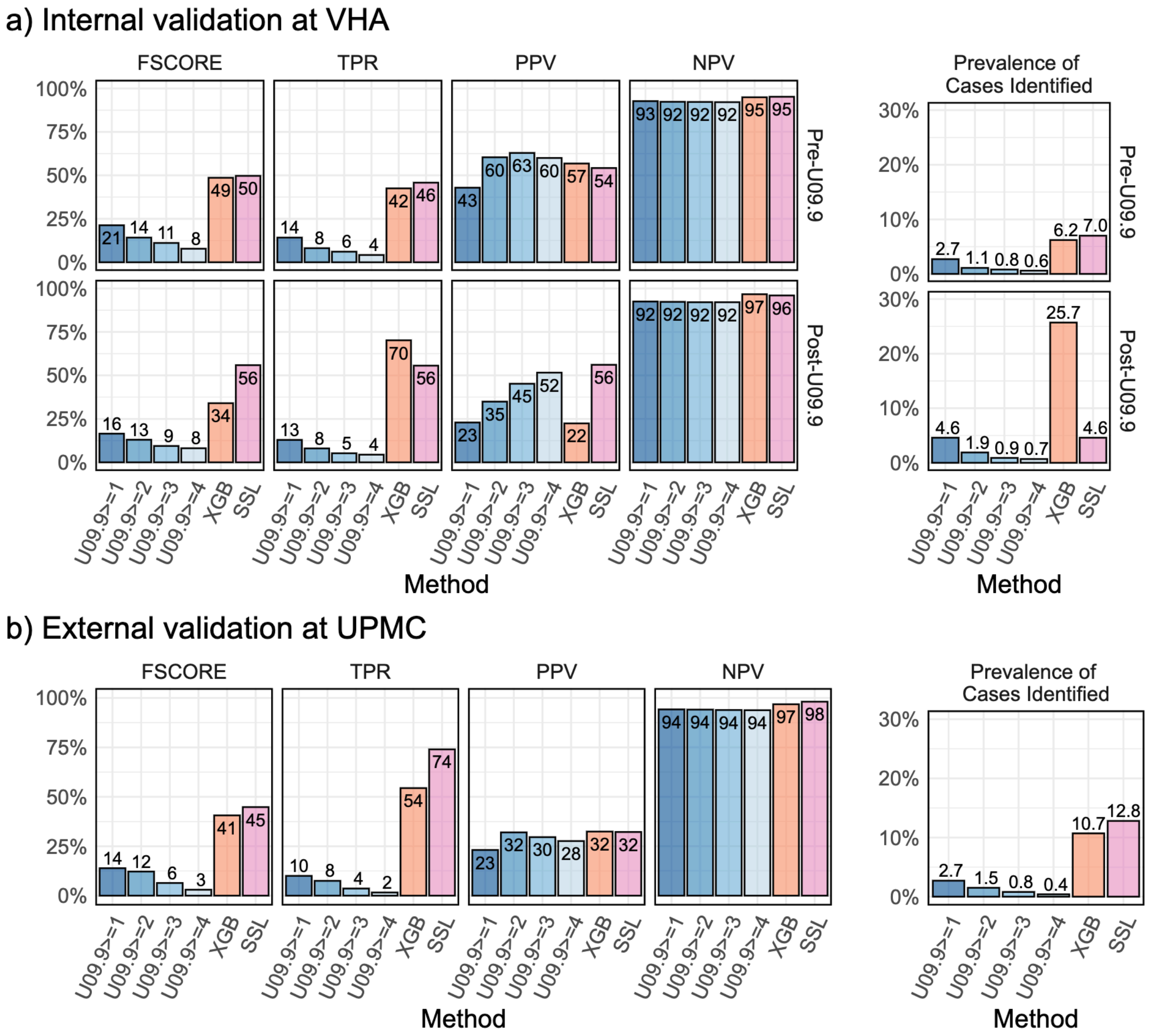

Supplementary Figure 3.

Pre- and post-infection healthcare utilization trend among patients identified as Long COVID cases vs. identified as Long COVID controls, stratified by COVID-19 variants.

The healthcare utilization data shown is stratified by different COVID-19 variants: Alpha, Delta, Omircron and others. Healthcare utilization is measured as the monthly total number of days with any PheCodes observed in the EHR. a) Pre- and post-infection healthcare utilization trend at VHA. b) Pre- and post-infection healthcare utilization trend at UPMC. Solid line - Long COVID cases, Dotted line - Long COVID controls, Red - Others, Green - Alpha, Blue - Delta, Purple - Omircron. VHA - Veterans Health Administration, UPMC - University of Pittsburgh Medical Center

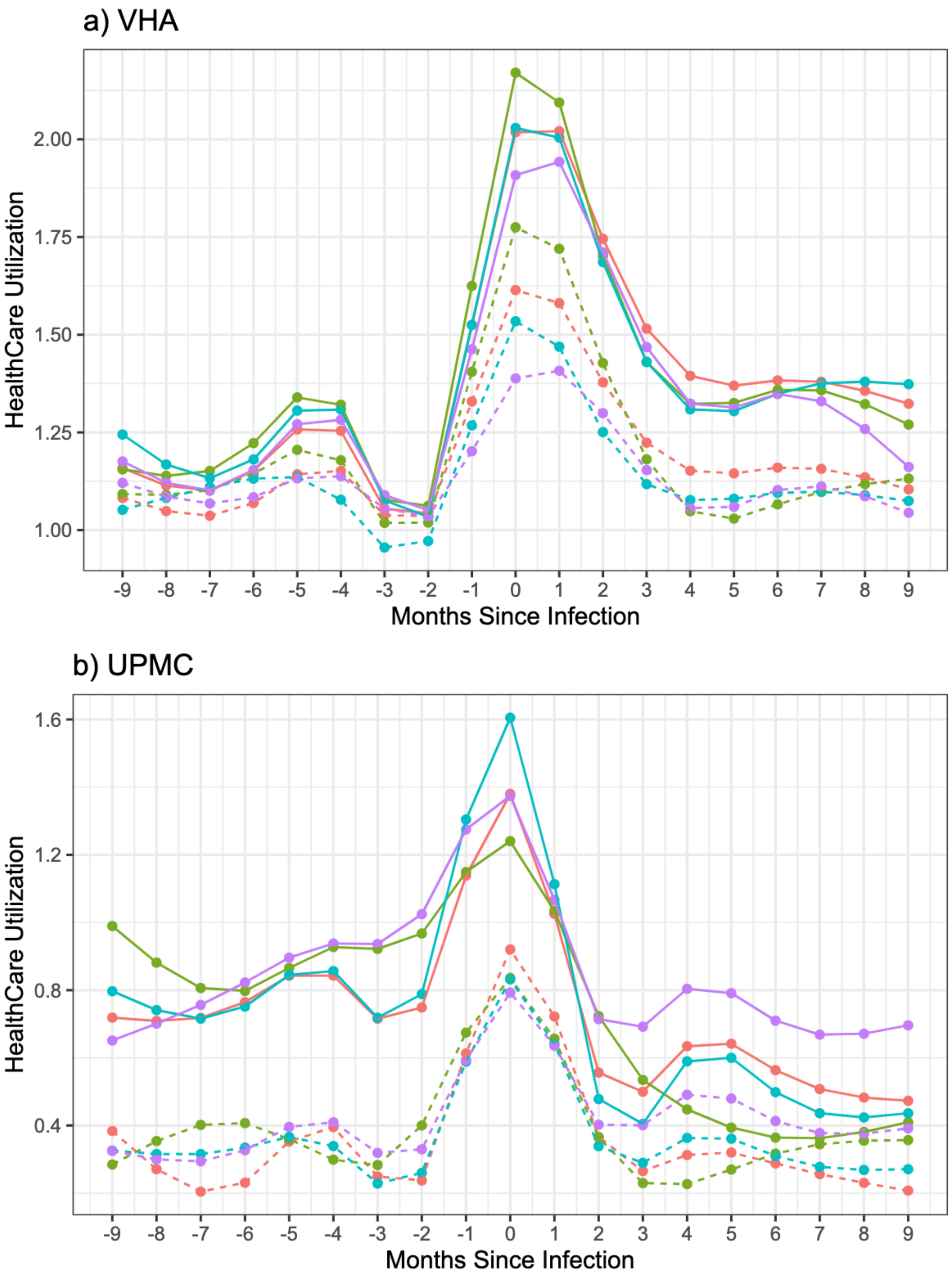
a) VHA
HealthCare Utilization
2.00
1.75
1.50
1.25
1.00
-9 -8 -7 -6 -5 -4 -3 -2 -1 0 1 2 3 4 5 6 7 8 9
Months Since Infection
b) UPMC
HealthCare Utilization
1.6
1.2
0.8
0.4
-9 -8 -7 -6 -5 -4 -3 -2 -1 0 1 2 3 4 5 6 7 8 9
Months Since Infection

Supplementary Figure 4.

Shapley feature importance values in supervised and unsupervised models at the VHA and UPMC.

Feature type is indicated by (n) for new onset counts and (m) for duration in months the feature was observed. For models with structured and unstructured data, data type of the feature is indicated by “PheCode” for coded feature, and “CUI” for NLP feature. a) Semi-supervised Shapley Values. b) Unsupervised Shapley values (top 10) for structured data only, pre-U09.9 period, inpatient model at VHA. c) Unsupervised Shapley values (top 10) for structured data only, pre-U09.9 period, outpatient model at VHA. d) Unsupervised Shapley values (top 10) for structured data only, post-U09.9 period model at VHA. e) Unsupervised Shapley values (top 10) for structured and unstructured data, pre-U09.9 period, inpatient model at VHA. f) Unsupervised Shapley values (top 10) for structured and unstructured data, pre-U09.9 period, outpatient model at VHA. g) Unsupervised Shapley values (top 10) for structured and unstructured data, post-U09.9 period, outpatient model at VHA. h) Unsupervised Shapley values (top 10) for pre-U09.9 model at UPMC. i) Unsupervised Shapley values (top 10) for post-U09.9 model at UPMC.

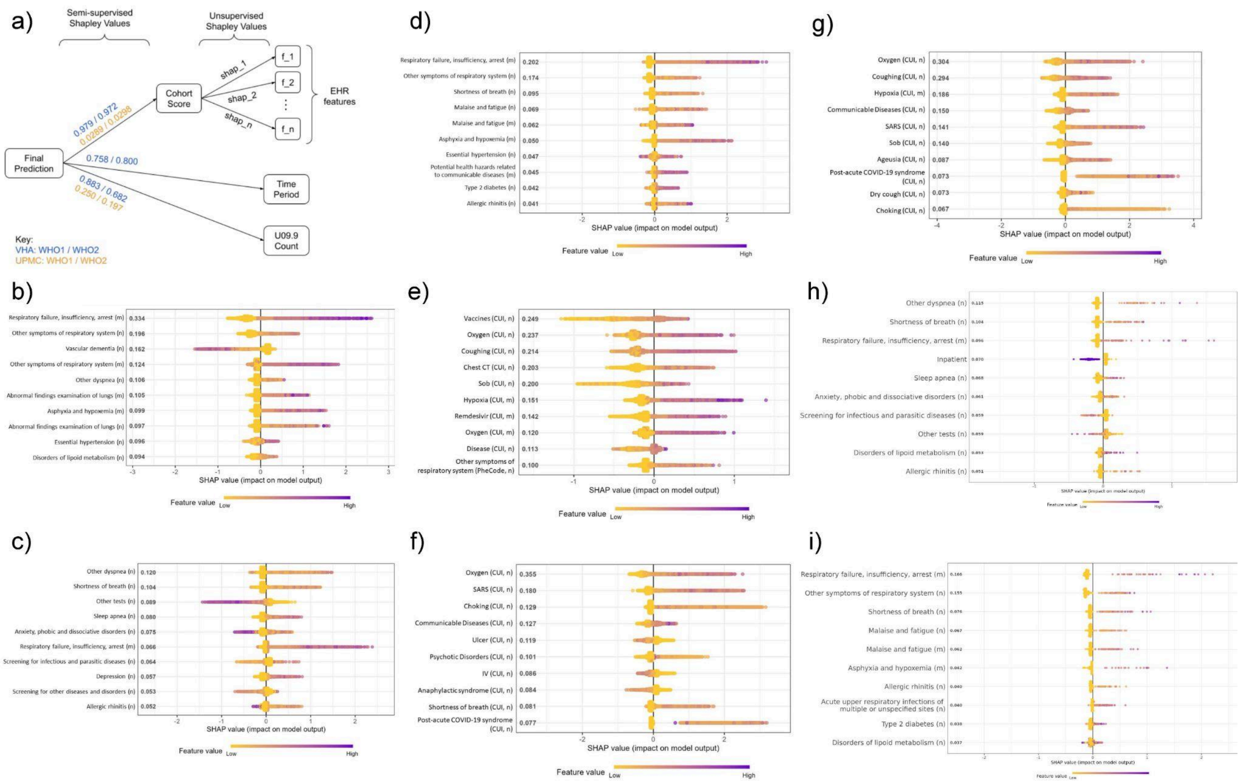

Supplement: Supplementary file 1 — Supplemental Material [file 41746_2025_1617_MOESM1_ESM.pdf]
